# An Adjusted Likelihood Ratio Approach Analysing Distribution of Food Products to Assist the Investigation of Foodborne Outbreaks

**DOI:** 10.1371/journal.pone.0134344

**Published:** 2015-08-03

**Authors:** Madelaine Norström, Anja Bråthen Kristoffersen, Franziska Sophie Görlach, Karin Nygård, Petter Hopp

**Affiliations:** 1 Department of Health Surveillance, Norwegian Veterinary Institute, Oslo, Norway; 2 Technische Universität München, München, Germany; 3 Department of Infectious Disease Epidemiology, Division of Infectious Disease Control, Norwegian Institute of Public Health, Oslo, Norway; Agricultural University of Athens, GREECE

## Abstract

In order to facilitate foodborne outbreak investigations there is a need to improve the methods for identifying the food products that should be sampled for laboratory analysis. The aim of this study was to examine the applicability of a likelihood ratio approach previously developed on simulated data, to real outbreak data. We used human case and food product distribution data from the Norwegian enterohaemorrhagic *Escherichia coli* outbreak in 2006. The approach was adjusted to include time, space smoothing and to handle missing or misclassified information. The performance of the adjusted likelihood ratio approach on the data originating from the HUS outbreak and control data indicates that the adjusted approach is promising and indicates that the adjusted approach could be a useful tool to assist and facilitate the investigation of food borne outbreaks in the future if good traceability are available and implemented in the distribution chain. However, the approach needs to be further validated on other outbreak data and also including other food products than meat products in order to make a more general conclusion of the applicability of the developed approach.

## Introduction

The impact of widespread foodborne outbreaks has increased in modern times. Many outbreaks of foodborne diseases that were once contained within a small community may now take place on a global scale [1–3]. This may be attributed to the globalisation of food production and changes in trade patterns [4].

A recent example of a large international outbreak is the European enterohaemorrhagic *Escherichia coli* (EHEC) outbreak in 2011. Investigation of the outbreak was challenging. Epidemiological studies and trace back studies identified bean sprouts as the source but the pathogen was never isolated from food samples [5,6].

The investigation of a foodborne disease outbreak usually includes epidemiological, environmental and food and laboratory investigations [7]. However, it may at times be difficult to identify the source using traditional epidemiological and microbiological methods. A person is usually unable to remember all the types of foods consumed during the incubation period of the infection [7], leading to recall bias. Therefore misclassification and missing information should be expected, which will increase the uncertainty of epidemiological studies. Furthermore, the laboratory examinations may not detect the causative agent as samples from the relevant product may not be available for testing, the pathogen may not be present in the samples taken due to heterogeneous distribution of the pathogen in the product, or the tests may not be sufficiently sensitive to detect the pathogen.

In 2006, Norway experienced a national outbreak of EHEC infections with 17 reported cases. The investigation was complicated and it took four weeks from when the alert was raised until contaminated fermented sausage was identified as the source of the outbreak [8]. Isolates with identical pulsed field gel electrophoresis (PFGE) and multi-locus variable number tandem repeat analysis (MLVA) profiles were isolated from the patients and the sausage products. However, all the isolates from the products lacked the *stx* genes [9]. Loss of *stx* genes during culturing and passaging has previously been described [10,11].

In outbreaks where the traditional methods fall short and the outbreak occurs over a longer period and/or in a larger geographical area, there is a need for additional tools to aid the outbreak investigations. Norström et al. [12] have suggested analysing the association between case and food product distribution to rank different food products as being the possible source of the outbreak. Doerr et al. [13] proposed a likelihood ratio approach independent of the underlying food distribution. Kaufman et al. [14] compared the likelihood ratio approach with a pairwise Spearman’s correlation method on simulated outbreaks using actual data on food distributions over a three year period without taking time into account. They found that a likelihood ratio approach best described their simulated outbreaks. The aim of our study was to examine the applicability of this likelihood ratio approach on real case and food distribution data from the Norwegian EHEC outbreak in 2006 and, if necessary, adjust the approach. Furthermore, the aim was to expand the approach by including time and space smoothing.

## Material and Methods

The likelihood ratio approach described by Kaufman et al. [14] was applied to real outbreak data from 2006. The methodology was further adjusted to include time, space smoothing and missing or misclassified information. All analyses were performed using the municipality as the geographical unit.

### Ethical statement

Human case data were provided by the Norwegian Public Health Institute. No informed consent was required because there were no ethical issues relevant to the study design and no individual-level analysis was performed. In this study the data have been de-identified prior to aggregation and analysis.

### Case Data

**Fig 1:**
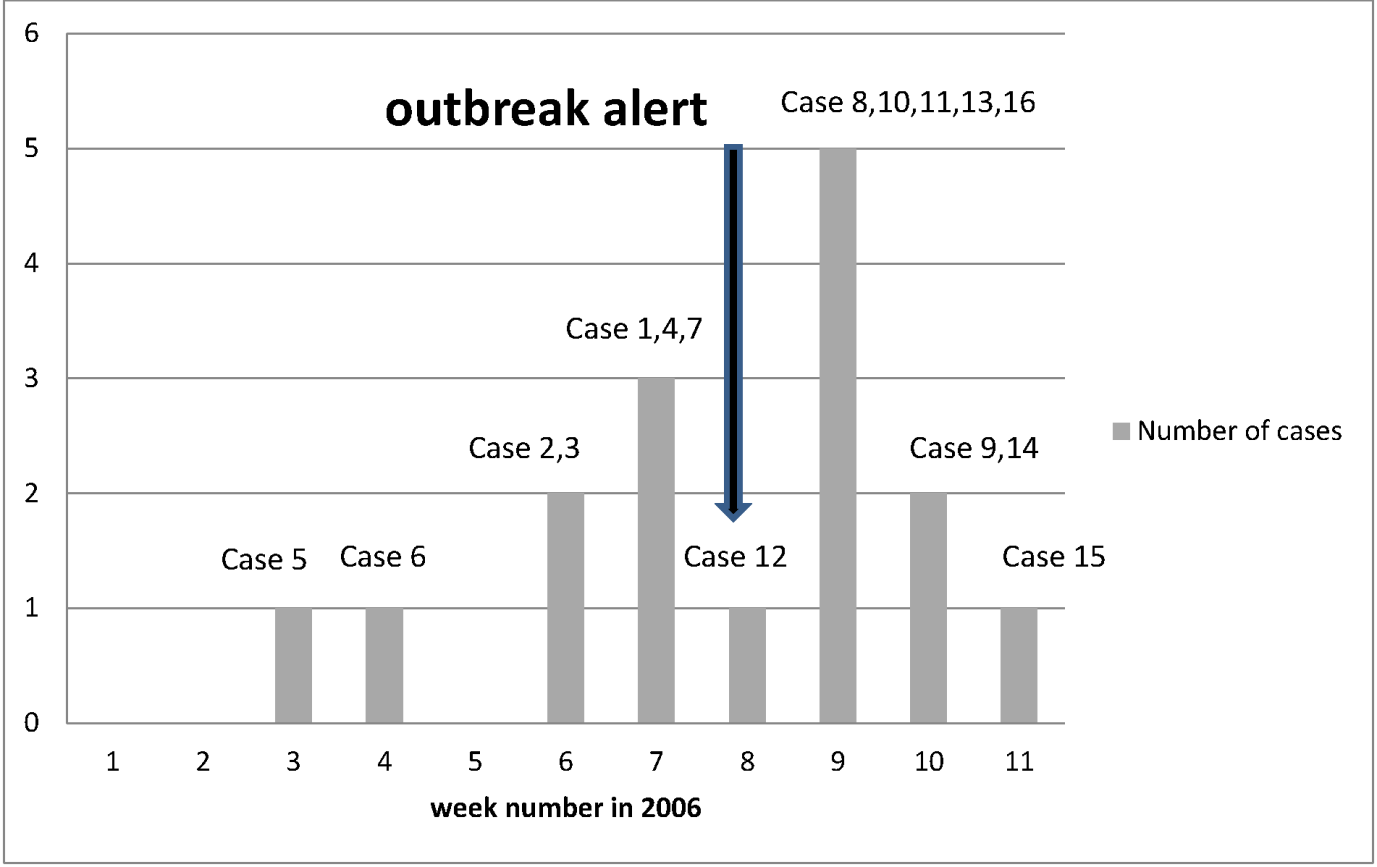
Weekly incidence of the Norwegian enterohaemorrhagic *Escherichia coli* O103 outbreak in 2006. The incidence is shown as the number of cases each calendar week. The sequence in which the cases were reported is illustrated by the numbering of the cases ‒ case 1, case 2 etc. The arrow indicates the outbreak alert. Later, case 5 was excluded from the outbreak based on laboratory examinations.

From January to March 2006 a total of 17 patients from 16 households were assigned to an outbreak of EHEC caused by enterohaemorrhagic *Escherichia coli* O103 in Norway [8]. Only the primary case in each household was included in this study; consequently, the study included 16 cases (Fig 1).

One of the cases (case 5) was later excluded from the 2006 outbreak as *E*. *coli* O103 was not laboratory-confirmed in faeces or blood and the patient had a positive serological reaction to *E*. *coli* O157. Since case 5 was assumed to be part of the outbreak during the investigation, the analysis was performed both with and without this case in order to assess how robust the approach would be for misclassification of cases.

The case exposure interval was based on the incubation period for the pathogen and defined as the time window in which the case could have been exposed to the contaminated food. The exposure interval will vary for different pathogens based on different incubation periods. For enterohaemorrhagic *E*. *coli* the exposure interval was defined as 1 to 14 days before the onset of illness [15–17].

The cases were assumed to primarily have consumed food purchased within their municipality of residence, but could have consumed food that had been bought outside their municipality of residence. Data from questionnaire surveys regarding the food purchase patterns or places where food had been consumed were available for most of the cases. Where this information was missing, the probable purchase pattern in neighbouring municipalities was used to impute missing data. Each case was assigned to one or several municipalities based on municipality of residence, neighbouring municipalities and information regarding the municipalities where food products could have been bought or consumed within the incubation period. For each case (*i*), each municipality (*m*) was given a weight (*w(i*,*m)* ∈[0,1]) where the sum of weights for each case was 1. One patient had been on holiday in another municipality during parts of his case exposure interval, and this municipality and its neighbouring municipalities were treated in the same way as the municipality of residence and its neighbouring municipalities. The weights were adjusted to the length of the stay in each municipality.

The cases were included in the analysis in the same order as they were reported (Fig 1); that is they were included as they would have been during a real outbreak investigation.

### Food product data

**Fig 2:**
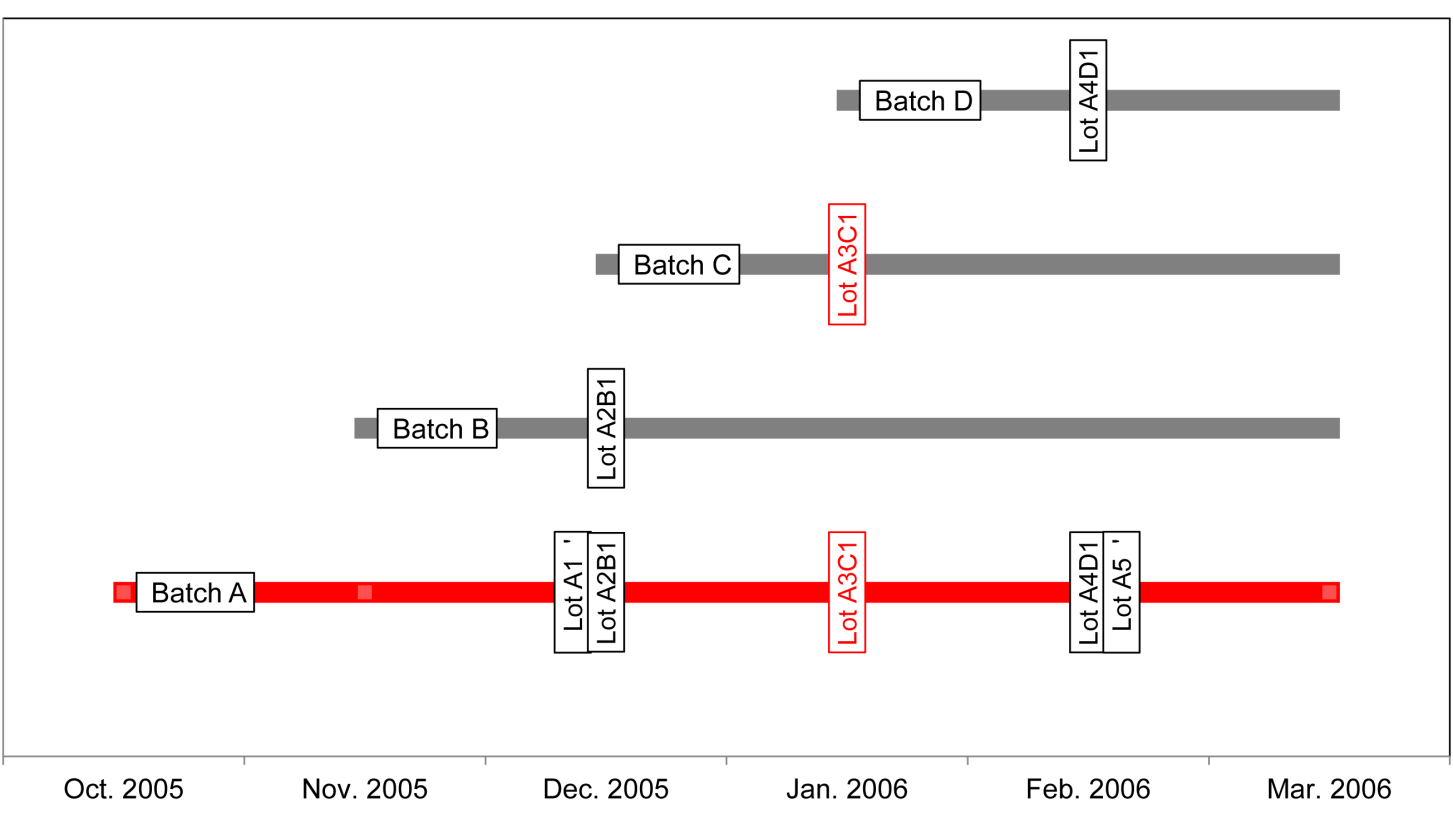
Schematic overview of most of the relationships between batches and lots from product X. Product X was the most suspected product. The batches and lots shown in this figure are those where most of the volume was sold during the outbreak. The food product units shown in red are the suspected batch and lot.

The outbreak in 2006 was attributed to contaminated fermented sausage with a median shelf life varying from approximately two to six months. A batch is the production of one type of sausages starting at a particular date made as one unit. This batch was fermented for several weeks. After curing, the sausage was sliced and put into consumer packages [18]. One type of sausage that was sliced on a particular date was designated as a lot. One batch could be the origin of one or more lots, and one lot could originate from more than one batch (Fig 2). We have designated product, batch and lot by the term food product unit and have been used this term in the following when appropriate.

During the outbreak investigation in 2006, distribution data for three different fermented sausages (hereby defined as product X, Y and Z) were collected. All deliveries of consumer packages of these products to individual retailers from 1^st^ December 2005 to 22^nd^ March 2006 were included, in total 53,551 deliveries. For all deliveries of product X, Y and Z, the following information was available: the product name, the package size, the weight and number of consumer packages, name and address of the individual retailers and the planned delivery dates. In addition, the expiry dates, the lot numbers and the batch IDs were available for the batches where the majority of the batches had been delivered during a period that could be connected to the outbreak. Only the food product unit ID, the municipality ID, the number of packages, the date delivered and the date expired have been included in the analysis.

Based on delivery and invoice address, each delivery was assigned to the municipality in which the receiving food retailer was located. For 19 (0.5%) of the retailers representing 0.4% of the consumer packages, the information was insufficient to assign to any municipality and the consumer packages in these deliveries was therefore only included in the total number of packages sold. For each delivery, the probable consumption interval was defined as the period from the delivery date to the expiry date. Missing values of the expiry date (1,682 deliveries, 3.1%) or unrealistically short expiry dates (1,905 deliveries, 3.6%) were imputed by adding the median period between delivery dates and expiry dates for the specific product to the delivery date given.

The number of consumer packages delivered was aggregated per date and municipality for each product, batch and lot. From 2006 there were three products, 51 lots and 85 batches, in total 139 different food product units that were included in the analyses. The number of municipalities to which the consumer packages belonging to a food product unit were delivered ranged from six to 425 municipalities, with a median value of 75 out of the total 430 municipalities. Not all deliveries could be attributed to a specific lot or batch as data on lot number and batch ID were difficult to acquire in 2006. These data were only collected for batches where a large proportion of the amount had been delivered within the outbreak period and was assumed to be a potential source of the outbreak. Consequently, for lots and batches that were considered unlikely as the source of the outbreak, lot and batch IDs were missing. However, these deliveries were still included at the product level constituting 56%, 51% and 51% of product X, Y and Z, respectively.

In 2006, epidemiological investigation found that one production lot in particular ‒ lot A3C1 of product X ‒ was repeatedly linked to the patients (Fig 2)[9]. Furthermore, when comparing the PFGE and MLVA profiles of *E*. *coli* O103 isolates from sausages and patients, the profiles most strongly related to the human cases were found in lot A3C1 and product X [9]. Therefore, lot A3C1, Batch A and Product X were considered the most probable food product unit responsible for the outbreak in this study [19].

In addition, data on food product units delivered to retailers in autumn 2009 and spring 2010 were used as control data. The manufacturer provided data of 91,679 deliveries for 16 products representing 275 lots (batch was not relevant for these data), in total 291 food product units. The information included the amount (in weight and number of consumer packages) delivered to individual retailers with the planned delivery date, the expiry date and the lot number. Only the food product unit ID, the municipality ID, the number of packages, the date delivered and the date expired have been included in the analysis. The distribution of the delivered consumer packages of a food product unit in the control data ranged from three to 390 municipalities with a median value of 145 out of the total 430 municipalities. For each food product unit belonging to the control data, the delivery dates were changed so that the product unit was delivered in a period that it could have caused the outbreak. The delivery dates was changed to the first possible date where at least one delivery could have been the cause of disease in the first case. Thereafter, new transformations of the data were made by increasing the delivery dates in steps of fourteen days until the last possible delivery date where a delivery could have been the cause of the outbreak. In total there were 2,487 transformed control datasets varying from two to 31 transformations for a particular food product unit, that were included in the analysis together with the original 2006 data sets.

### Statistical approach

Kaufman et al. [14] describe a likelihood ratio approach when each case *i* occurs in a given postal zone *m*_*i*_. Time of delivery and exposure is not included in the model. To calculate the likelihood ratio they first find the frequency (or probability) that a product *n* is consumed in the postal zone *m* of interest for case *i*, i.e. postal zone *m*_*i*_, by calculating all sales *sales*(*n*,*m*_*i*_) that have occurred in postal zone *m*_*i*_ and dividing by the total sale of product n.

$$f\left(i,n\right)\;=\;\frac{sales\left(n,m_{i}\right)}{\sum_{m\epsilon M}sales\left(n,m\right)}$$

The likelihood ratio of product *n* was then found to be proportional to the product of frequencies for each case *i*:

$$L\left(n\right)\propto \prod_{i=1,\ldots ,I}f\left(i,n\right)$$

Thereafter the maximum likelihood over all of products is found, so that the likelihood ratio could be calculated as *L*(*n*) divided by max(*L*(*n*)).

### Adjustment of the likelihood ratio approach

In this study we included both a geographical distribution for each case and a time perspective in the likelihood ratio approach. Each case had an accompanying exposure interval and each delivered consumer package an accompanying consumption interval. For each case, *i*, the probability that each product, *n*, was consumed in a municipality, *m*, f(*i*, *n*, *m*), was calculated based only on deliveries with a probable consumption interval that overlapped the case exposure intervals.

The deliverables included for each case were defined as *sales*(*i*, *n*, *m*), where *sales*(*i*, *n*, *m*) was the number of packages sold of product *n* in municipality *m* that had a consumption interval overlapping with the exposure interval of case *i*. In the denominator the overall sum of municipalities, *mϵM*, of all products that were delivered before the end of the last exposure interval was included, *sales2*(*i*, *n*, *m*). This was defined as all sales that have possibly occurred before the end of the last exposure interval when case *i* was reported. The probabilities of consumption of each product were then calculated as

$$f\left(i,\;n,\;m\right)\;=\;\frac{sales(i,\;n,\;m)}{\sum_{m\epsilon M}sales2\left(i,\;n,\;m\right)}$$

where *M* includes all 430 municipalities in Norway and an additional artificial municipality that was assigned the deliveries for which the information on municipality was missing. For each *i* and *n* the sum of *f*(*i*, *n*, *m*) over all municipalities *m* will be less than or equal to 1.

Each case had accompanying municipality weights, *w*(*i*, *m*) as described in the section Case data. These weights were used together with the case corresponding to municipality frequencies of consumption of each product *f*(*i*, *n*, *m*) to calculate a case-product frequency *f*(*i*, *n*):

$$f\left(i,n\right)=\sum_{m\epsilon M}w\left(i,m\right)\;*\;f(i,n,m)$$

The likelihood that product *n* was the source of the outbreak will then be proportional to the case-product frequency for each case *i* that has occurred. If there have been a total of *I* cases the likelihood will be proportional to

$$L\left(n\right)\propto \prod_{i=1,\ldots ,I}f\left(i,n\right)$$

Many of the case-product frequencies *f*(*i*,*n*) will be zero. To handle a scenario where a case is wrongly included in the outbreak or where a municipality weight associated to a case is incorrectly assigned to zero, a background probability equal to a given small number, *ε*, is added to all case-product frequencies to calculate an adjusted likelihood that will be proportional to

$$L_{adjust}\left(n,I\right)\propto \prod_{i=1,\ldots ,I}\left(f\left(i,n\right)+\varepsilon \right)$$

Let *L*_*adjust*_ (*I*) be a vector of all proportionally adjusted likelihood *L*_*adjust*_ (*n*,*I*). By dividing all elements in *L*_*adjust*_ (*I*) by the maximum element of *L*_*adjust*_ (*I*) an adjusted likelihood ratio is obtained. To ensure that the adjusted likelihood ratio does not diverge much from the actual likelihood ratio, we chose to use a small number comparable to the smallest non-zero frequencies calculated.

The results were visualised by box plots showing the distribution of the adjusted likelihood ratios according to the number of reported cases included in the analyses. The calculation of a likelihood “ratio” does not affect the ranking order and is actually applied to normalize the calculated scores which are useful for the visualization purposes.

The product, batch and lot that were identified as the most probable food product units responsible for the outbreak were indicated in the box plot to visualise their ranking. Rank numbers above 30 were not shown.

### Scenarios

The basic scenario (Scenario A) included case 5 and the consumption and exposure intervals in the analyses. In addition, the analyses were run without case 5 (Scenario B), without the consumption and exposure intervals (Scenario C), and without both case 5 and the consumption and exposure intervals (Scenario D). A sensitivity analysis of *ε* was performed by comparing the results based on different values of *ε*.

### Data management and analysis

The raw data were received as Excel files and were prepared for analysis by using SAS-PC System v 9.1.3 for Windows (SAS Institute Inc., Cary, NC, USA). The analyses were performed using R version 3.02 for Windows [20].

## Results

**Fig 3:**
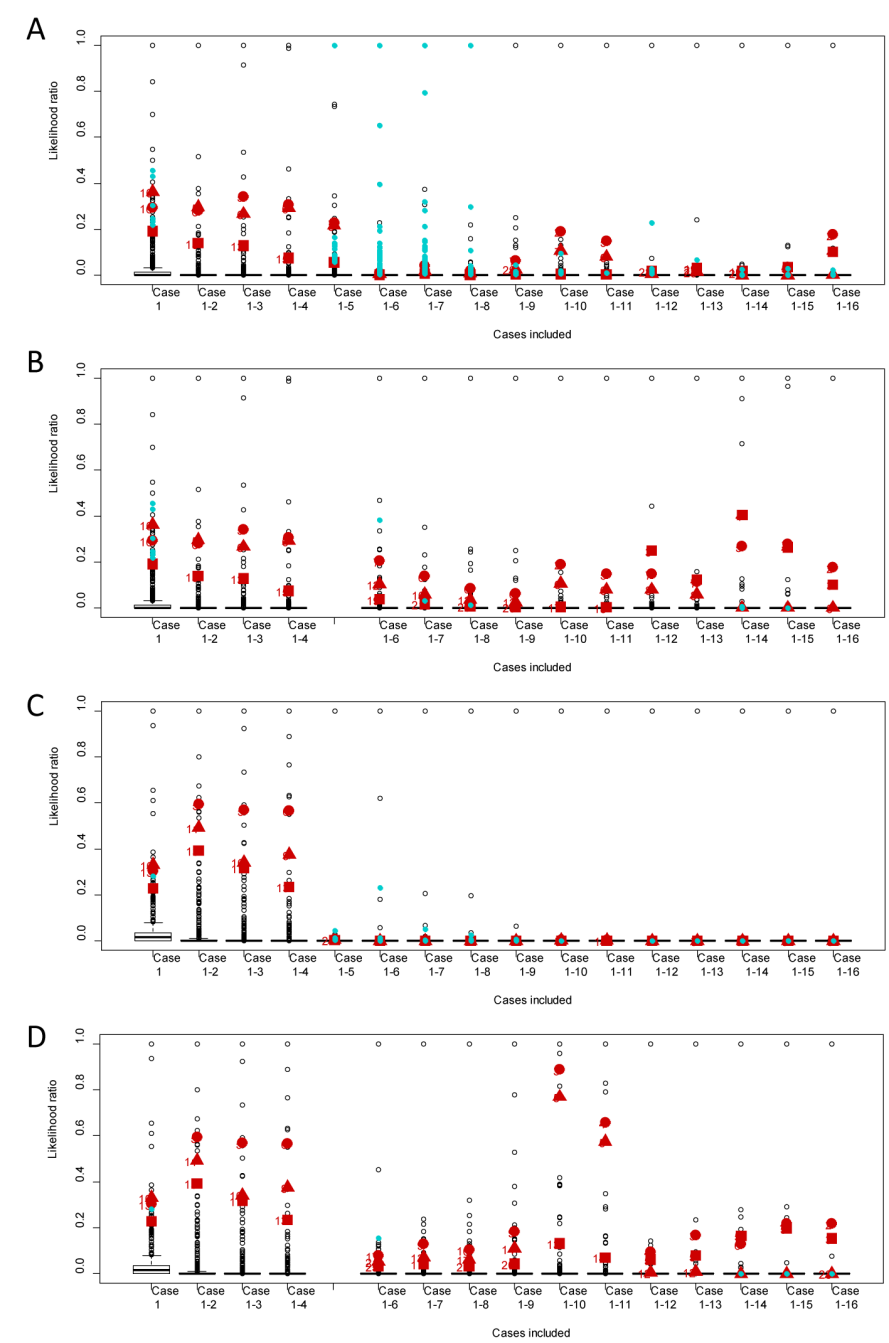
Boxplots of the likelihood ratio results for all 2,626 food product units in the different scenarios. A: basic scenario, B: scenario B, excluding case 5, C: scenario C, excluding consumption and exposure intervals and D: scenario D, excluding case 5 and consumption and exposure intervals. The x axis specifies the included cases from case 1, case 1 and 2 etc. up to case 1 to 16 included in the analyses. The boxplot shows the median, 25 and 75 percentiles within the box. The upper and lower whiskers indicate the area between which the data would have been distributed given Gaussian distribution. The dots below or above the whiskers indicate outliers or extreme values. The product (square), batch (circle) and lot (triangle) which were identified as the most probable food product units responsible for the outbreak are indicated in red with their ranking on the left side, rank numbers above 30 are not shown. The blue circles indicate control data based on the food product units delivered in 2009 and 2010 and transformed to 2006 data that had a likelihood ratio greater than the worst suspected food product unit.

The product, batch and lot identified as the most probable source (product X, batch A and lot A3C1) of the outbreak was among the ten most likely food product units after two cases had been included in the basic scenario (Fig 3A). However, the ranking of these suspected food product units decreased when case 5 and subsequently case 6 were included. When including these cases (5 and 6), many products from the control data were also ranked among the most likely product units. After including the nine first reported cases, the suspected product, lot or batch were again among the ten most likely food product units. By the further inclusion of case 12 the suspected food product units achieved a lower likelihood ratio. This low ranking persisted until case 15 was included, when both the batch and the product were among the five food product units with the highest likelihood. The food product unit that obtained the highest likelihood after 16 cases was lot A1 (Fig 2) originating from the same batch A as the suspected lot A3C1.

In the scenario B, (excluding case 5, Fig 3B), at least one of the suspected food product units was among the ten most likely food product units from the inclusion of case two, and remained at this ranking level when the other reported cases were included. Furthermore, fewer food product units from the control data came out with a higher likelihood than the most suspected food product units. The same lot (A1) as in the basic scenario was the most likely food product unit after the inclusion of case 14 as well as later cases.

In the scenario C (excluding consumption and exposure intervals, Fig 3C), the ranking achieved for the first four cases did not differ noticeably from the basic scenario (Fig 3A). However, when case 5 was included, the likelihood of the suspected product, lot and batch dropped below the ten highest ranked food product units. By the further inclusion of case 6, the highest suspected food product unit had 700 food product units with a better likelihood. It was not until ten cases were included that one of the most suspected food product units again was among the ten with the highest likelihood ratio.

In the scenario D (excluding both case 5 and consumption and exposure intervals, Fig 3D), the results were comparable to the scenario B (Fig 3B). The consumption and exposure intervals for the suspected food product units and case 5, case 6 and case 12 did not overlap in any municipalities, consequently, all case-product frequencies were zero for these cases. In total, 67% of the case-product frequencies calculated per municipality were zero. The analyses above (Fig 3) were made with a background frequency equal to 10^−5^, which corresponds to the lower 0.25 percentile of the non-zero case-product frequencies calculated. The smallest non-zero case-product frequency calculated was 9*10^−7^. We tested the use of a background frequency equal to 10^−6^ (S1 Fig) which was too small to handle the inclusion of cases 5 and 6. The ranking of the likelihood ratio for the most suspected food product unit dropped to 417 and a rank number below 50 was not achieved until case 15 was included (rank number 47). With a higher background frequency the suspected food product units obtained better rankings and the inclusion of case 5, 6 and 12 did not influence the ranking substantially (S1 Fig). The results obtained using the original likelihood ratio approach, are presented in S2 Fig for comparison.

## Discussion

This study showed that the adjusted likelihood approach applied to data from a small EHEC outbreak is promising to help identifying possible sources of food born outbreaks. However, the approach needs to be used in combination with microbiological and epidemiological investigations. The fact that the suspected food product units obtained a high ranking, among the 20 highest, after only a few cases indicates that the approach may be useful in the early phase of an outbreak investigation.

The approach used in this study is a modification of a likelihood ratio approach previously developed and applied to simulated outbreak data [14]. We have developed the likelihood ratio approach further by including time; i.e. consumption and exposure intervals, smoothing of case consumption area and a background frequency ε to account for uncertain information that will often be found in real data.

By including time, we were able to refine the likelihood ratio approach to account for the fact that a real outbreak will be limited in both space and time. If time is not included, the approach will primarily identify products based on the general distribution pattern of the food products. By including time, one may be able to identify specific batches and lots that have been contaminated. We used a 1 to 14 day incubation period and data on known delivery and expiry dates to identify the food product units that could have been the source of the outbreak. There might have been a larger variation in incubation time. Food products might also have been consumed after the expiry dates. However, for the studied outbreak and at least when the incorrectly allocated case (case 5) was included, our analyses showed that in order to distinguish between the included food product units, the inclusion of consumption and exposure intervals were necessary. This was clearly seen when comparing the results from the basic scenario with Scenario C in which the consumption and exposure intervals were excluded. The food product unit that achieved the highest rank in Scenario C was distributed to 161 municipalities and, because the consumption and exposure intervals were not included, this food product unit achieved a high rank throughout the analyses.

In this study we used municipality as the unit of concern. However, purchase of food is usually not limited to the residence municipality only. The household will purchase food in nearby municipalities as well as other places where household members have been working or holidaying etc. We used information on purchase patterns for each case collected by questionnaires to smooth the case data geographically. If such information was lacking or scarce, we imputed weights for the cases to neighbouring municipalities based on knowledge of the infrastructure of the municipalities. Detailed information on purchases made by the cases may not always be available, either because there is a delay in information-gathering or due to confidentiality issues. Therefore the development of other approaches for geographical smoothing may be beneficial. One approach could be based on Huff’s gravity model for trade area analysis [21] as suggested by Kaufman et al. [14]. If the exact coordinates of the residence are known, it might be possible to apply other criteria for accessibility of food products based on distance to retail shops in rural vs. urban areas as applied in the study by Hashemi Beni et al. [22]. Most of the 20 food product units with the highest likelihood ratio originated from the 2006 data set except the time points where the proportion of misclassified cases included were above or equal to 20%. These consisted of fermented sausages produced in a single processing plant and with a shelf life of several months. The control data consisted of a range of different meat products with a large variation in distribution pattern and usually a shorter shelf life than the fermented sausages. The good discrimination between these products that was achieved by the likelihood ratio approach is probably due to both the spatial and time distributions.

The inclusion of a background frequency, ε, for every product ensured that the likelihood ratio did not drop to zero because of the inclusion of erroneously allocated cases or missing or incorrect information. In an outbreak situation, we expect that recall bias as well as misclassified and missing information will be a problem, in particular in the early phase of the outbreak. In the 2006 outbreak, none of the suspected food products had been delivered to any municipalities linked to cases 6 and 12. Furthermore, one case (5) was initially included in the outbreak based on clinical signs but later found not to be part of the outbreak. In other outbreak situations there might be more cases that are not linked to contaminated products and the performance of the methodology in such situations is unknown. However, the approach applied on our data indicated that it was able to handle misclassifications up to 19%.

By using the likelihood ratio approach without a small background frequency for every case, the suspected product, batch and lot would obtain a likelihood ratio estimate equal to zero when data from these cases (5, 6 or 12) were included in the analysis. Consequently, the original approach without a background frequency ε as described by Kaufman et al. [14] would not identify these suspected food product units as being among the products that had most likely caused the outbreak.

The higher the background frequency, the further away the adjusted likelihood ratio will be from the true likelihood ratio. Therefore, a low background frequency should be used. The size of the background frequency might not be easy to define, but in our analyses an ε at the lower 0.25 percentile of the non-zero case-product frequencies was sufficient to identify the suspected product units by the adjusted likelihood ratio approach.

The suspected food product units had a high likelihood ratio, which support that contamination of these products might be the cause of the outbreak. Isolates with identical PFGE and MLVA profiles were isolated from the patients and these products. Although, all the isolates from the products lacked the stx genes that does not exclude them from being the source of the outbreak as loss of stx genes during culturing and passaging previously has been described [10,11].

During an outbreak investigation we suggest that the results from the adjusted likelihood ratio approach should be interpreted with caution. We recommend that the adjusted likelihood ratio approach should be used primarily for selecting a large numbers of products for epidemiological and laboratory investigations. Used in combination with other methods, we believe that the adjusted likelihood ratio approach will be a useful tool to rapidly target the laboratory analyses of the most likely sources, as well as to rule out less likely sources.

In this study, the data was aggregated on lot, batch and product level for a single product unit. However, in a foodborne disease outbreak the problem may not be the single product but a contaminated ingredient that has been used in several different products or a production plant or equipment within a production plant that has contaminated several products during a particular time period. Therefore, aggregation of data at the level of ingredient, the production site and production machines would be needed. If such information is available, the approach presented should also be able to handle such data.

In situations where a food borne outbreak is caused by a pathogen with a high background frequency, such as for example *Salmonella* enteritidis in some countries, there would be no distinctions between the cases caused by the outbreak and the cases occurring as a result of the endemic situation. In such situations, this approach would not be applicable.

There are some prerequisites that have to be fulfilled for the adjusted likelihood ratio approach to apply. Firstly, the case definition needs to be clear; however the adjusted likelihood ratio approach was able to handle 19% misclassified cases in our dataset.

In an outbreak situation, all suspected cases need to be included in the pre-analyses and then later excluded if laboratory diagnoses show that they are unrelated to the outbreak strain. Secondly, for each food product unit, information regarding all deliveries per geographical unit is needed. Thirdly, the residence or place of infection for each case (geographical unit) is needed as well as information or assumptions regarding the geographical unit in which each case has been purchasing or consuming her/his food. In this study, the food manufacturer was able to provide sufficient data to perform the analyses. However, the food distribution chain might involve several steps and the tracing and tracking of a product in the delivery chain are often complicated [23]. Therefore, to use this tool in future outbreak investigations, good traceability systems need to be available and implemented in the distribution chain.

## Conclusion

The adjusted likelihood ratio approach is promising and can be a useful tool to assess and prioritise further investigations of suspected food products during a foodborne outbreak. The adjustment of the likelihood ratio approach by Kaufmann et al. was necessary in order to identify the most suspect products in the data from the HUS outbreak in Norway 2006. However, the approach needs to be further validated on other outbreak data and also including other food products than meat products in order to make a more general conclusion of the applicability of the developed approach. The use of the approach is dependent on availability of relevant data, in particular food product unit distribution, and traceability systems for food products need to be improved. During an outbreak investigation we suggest that the results from the adjusted likelihood ratio approach should be interpreted with caution and together with epidemiological and laboratory investigations.

## Supporting Information

S1 Fig Boxplots of the likelihood ratio results for all 2,626 food product units using different background frequency epsilon in the basic scenario (A). (PDF)

S2 Fig Boxplot of the likelihood ratio results for all 2,626 food product units using the original likelihood ratio approach. (PDF)

S1 File cases.txt Anonymised case files; Case = a number of the case as it were reported during the outbreak, “Municipality” = Identifier of the Municipality. “Index” = the weight of the case and “firstdate” = the date of disease onset.
(TXT)

S2 File casesNoGeographical Smoothing. Anonymised case files; Case = a number of the case as it were reported during the outbreak, “Municipality” = Identifier of the Municipality. “Index” = the weight of the case (here equal 1 for all cases as no weighting are included) and “firstdate” = the date of disease onset.
(TXT)

S3 File Product files. Anonymised files of the distribution of each food product unit included in the analysis. Variables are “Municipality” = Identifier of the Municipality,”NumPackages” = Number of consumer packages delivered;”Date_delivered” = The date the food product unit has been delivered;”Date_expired” = The date given where food product unit have expired.
(ZIP)

S4 File R Code Adjusted Likelihood Ratio Approach. (TXT)
