# Supplementary material for: An Adjusted Likelihood Ratio Approach Analysing Distribution of Food Products to Assist the Investigation of Foodborne Outbreaks
Source: PLoS One. 2015 Aug 3;10(8):e0134344. doi: 10.1371/journal.pone.0134344 (PMC4523185; doi:10.1371/journal.pone.0134344)

S2 Fig. Boxplot of the likelihood ratio results for all 2,626 food product units using the original likelihood ratio approach.

The x axis specifies the included cases from case 1, case 1 and 2 etc. up to case 1 to 16 included in the analyses. The boxplot shows the median, 25 and 75 percentiles within the box. The upper and lower whiskers indicate the area between which the data would have been distributed given Gaussian distribution. The dots below or above the whiskers indicate outliers or extreme values. The product (square), batch (circle) and lot (triangle) which were identified as the most probable food product units responsible for the outbreak are indicated in red with their ranking on the left side, rank numbers above 30 are not shown. The blue circles indicate control data based on the food product units delivered in 2009 and 2010 and transformed to 2006 data that had a likelihood ratio greater than the worst suspected food product unit.

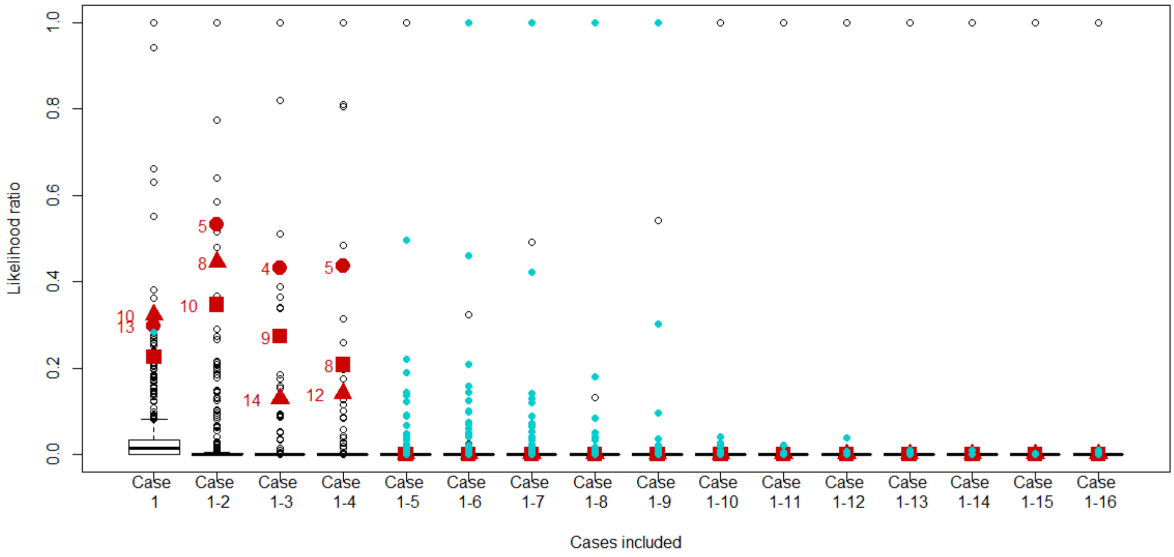

Supplement: S2 Fig — (PDF) [file pone.0134344.s002.pdf]
